# A case of cavernous hemangioma of the infratemporal fossa causing recurrent secretory otitis media

**DOI:** 10.1016/j.bjorl.2021.03.001

**Published:** 2021-03-26

**Authors:** Edoardo Covelli, Valerio Margani, Guido Trasimeni, Chiara Filippi, Giorgio Bandiera, Simonetta Monini, Maurizio Barbara

**Affiliations:** a Sant’Andrea Hospital, NESMOS Department, Medicine and Psychology, ENT Clinic, Rome, Italy; b Sant’Andrea University Hospital, NESMOS Department, Otolaryngology Clinic, Rome, Italy

## Introduction

Secretory otitis media is defined as the presence of fluid in the middle ear space causing aural fullness and hearing loss secondary to Eustachian tube obstruction or incomplete resolution of acute otitis media.^1^ Most of cases are self-limiting with recovery within 2/3 weeks, while others require medical management. Diagnosis includes otoscopy and the presence of typical aural symptoms. Tympanometry is mandatory to confirm the presence of fluid in the middle ear and to evaluate Eustachian tube function. In more severe cases, audiometry, temporal bone computed tomography (CT) and magnetic resonance imaging (MRI) can be used to evaluate associated complications. Every patient with unilateral middle ear effusion should undergo nasopharyngoscopy to assess the nasopharyngeal space.^2^ When findings on both examinations are normal, many adult patients undergo additional medical managements or ventilation tube insertion.^3^ This approach may be successful in patients with typical recurrent middle ear effusion and normal nasopharyngoscopy; however, Eustachian tube narrowing due to occult lesions may be missed if further investigation is not carried out. Expansive lesions at the level of pterygopalatine fossa may cause Eustachian tube compression with subsequent tube dysfunction with clinical findings of recurrent unilateral secretory otitis media. In this paper we report a case of cavernous hemangioma of the infratemporal fossa causing recurrent secretory otitis media.

## Case report

**Figure 1:**
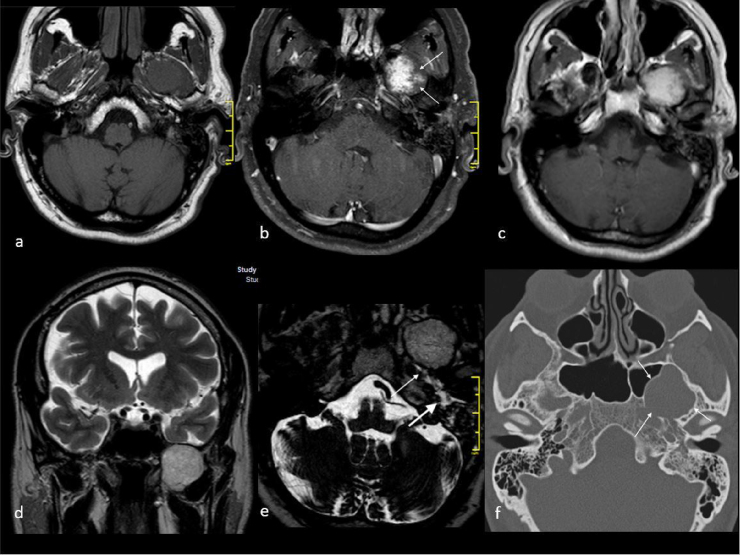
(a) Ax T1 image shows homogeneous iso-signal of the lesion to muscle. Early post gd injection on B image (ax T1 3D fat sup) shows incomplete enhancement (arrow). A few minutes after gd injection on c image (Ax T1 se) the enhancement is complete, homogeneous. On T2 wi (d image, coronal turbo T2) the lesion shows high homogenous signal. On Ax T2 3D image (e) it’s clearly evident the high signal of retention material in middle ear and in the tuba (arrows). CT bone image (f), the bone is remodeled by lesion expansion (arrows) without erosion, typical aspect for long standing lesion.

**Figure 2:**
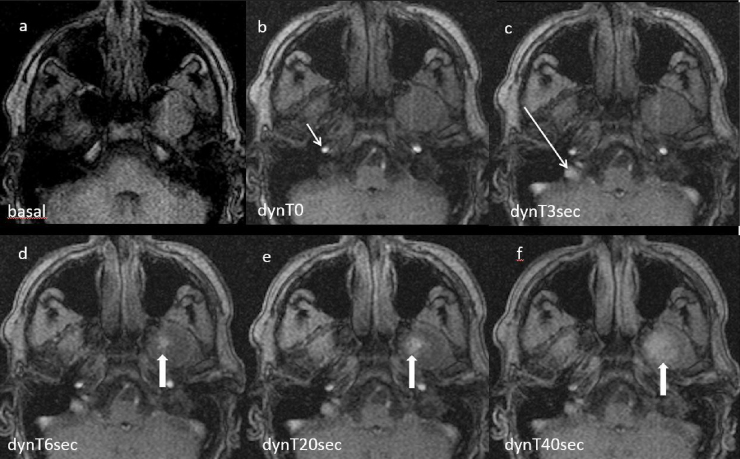
(a) Sequential same slice during gadolinium injection on turbo fast FFE sequence showing progressive lesion enhancement starting late during the venous phase (arrow in d-e-f). (b) The arrow shows ICA enhancement in the arterial phase. (c) The arrow shows sigmoid sinus enhancement in venous phase with no further lesion enhancement.

A 55 years old, otherwise healthy man presented with a history of hearing loss and fullness in the left ear. Otoscopy showed a hyperemic tympanic membrane with fluid build-up behind it in the left ear, while the right ear was normal. Cranial nerve examination did not show any abnormality except for a slight hypoesthesia of the facial area innervated by the second trigeminal branch. Audiometry showed mild sensorineural hearing loss on the right side and moderate mixed hearing loss on the left side. Tympanometry was type “As” on the right side and type “B” on the left side. Nasopharyngoscopy did not reveal abnormalities in the nasopharynx, hypopharynx and at the level of the glottis. The clinical findings were therefore suggestive for serous otitis media and a treatment with systemic steroids, antibiotic and decongestant nasal spray was established. The patient underwent a myringotomy with insertion of transtympanic drainage tube. Three months later, he presented with vertigo associated with nausea and neurovegetative symptoms, with left side hearing loss not associated with postural changes. Vestibular examination showed grade 2 left-beating spontaneous nystagmus with a positive Romberg test on the left side. The clinical findings were consistent with a diagnosis of vestibular neuritis and a brain MRI scan was then performed (Figure 1, Figure 2). The scan showed the presence on the left side of a 3.7 × 3 × 2.5 cm solid mass in the infratemporal masticatory space adhering with the oval foramen and reshaping the middle cranial fossa floor, causing a slight contact on the temporal lobe with erosion of the greater sphenoid wing. Medially it made contact with Meckel’s cave and lateral aspect of sphenoid sinus. Postero-medially it occupied the Eustachian tube anatomic region, in association with signs of fluid buildup. Laterally it was situated between lateral and medial pterygoid muscles. It is hyperintense in T2w images, hypointense in T1w images; dynamic scan shows a late enhancement with centrifugal pattern. The pterygopalatine fossa was normal. A CT integration was then performed. It showed a middle cranial base mass which reshaped surrounding bone structures, with obliteration of left oval foramen. This finding caused reshaping of the superior aspect of homolateral pterygoidal processes, extending downwards in the left masticatory space. The patient gave written informed consent to the surgery, and the study was conducted in accordance with the provisions of the Declaration of Helsinki. The patient underwent endoscopic transnasal resection of the lesion. A medial, type B maxillectomy was performed, and inferior ethmoidal resection was carried out to reveal the orbito-maxillar space. Sphenoidotomy was then performed. The posterior wall of maxillary sinus was removed, and the pterygopalatine infratemporal spaces were opened. The lateral sphenoid recess was exposed, and the second trigeminal branch was identified. The lesion caused a bulge on the floor of left lateral sphenoid recess and seemed to be originating from the third trigeminal branch. The internal component of the lesion was then debulked and the capsule was dissected from mandibular root and from pterygoid insertions.

The capsule was then dissected superiorly, where the lesion encountered the middle cranial fossa dura. The lesion was removed. Histopathological examination reported a CD31+ hemangioma of the II trigeminal branch, with benign features. Brain MRI performed after 6 months did not show any evidence of disease recurrency.

## Discussion

Unilateral middle ear effusion may result from Eustachian tube compression or invasion rather than from physiologic dysfunction. Gacek identified the four levels of potential Eustachian tube compromise as being the lumen of the Eustachian tube, the nasopharynx, the infratemporal fossa and the petrous apex.^4^ When initial evaluation and nasopharyngoscopy are normal, most otolaryngologists actuate medical management and, if it is unsuccessful, ventilation tube placement is generally offered.^3^ Main diseases that originate in this site are adenocarcinomas, adenoid cystic carcinoma, liposarcoma, hemangioma, teratoma, lymphoma, leiomyosarcoma and undifferentiated sarcoma.^5^ Hemangiomas are benign vascular tumors and the most common benign tumors of the head and neck region.^6^ The major subtypes are the capillary, cavernous and mixed. In the majority of the cases, the cavernous type is associated with the lateral wall of the nasal cavity or with the inferior turbinate. Cavernous hemangiomas are uncommon congenital malformations, and they may manifest during adulthood and do not involute spontaneously.^7^ Clinical examination and nasopharyngoscopy are usually normal, therefore the diagnosis is completed with imaging. While CT imaging might not adequately identify an underlying mass lesion,^8^ hemangiomas present an iso- or hypointense signal on T1-weighted MRI and an hyperintense signal on T2-weighted imaging. Also, these tumors show intense contrast enhancement.^9^ Hemangiomas are rarely found in infratemporal or pterygopalatine fossa. A review conducted on 79 patients affected by middle ear effusion showed that 63.3% of the patients were affected by malignant tumor, 32.9% by benign tumor and 3.8% by aneurism of the internal carotid artery. Of these, histopathologic analysis never showed evidence of hemangioma.^8^ In another study similar to ours, Carlstrom et al. in 2017, reported a case of an occult neurofibroma of infratemporal fossa causing unilateral tinnitus and middle ear effusion.^10^ In 2019 Sahin et al. reported the only case of cavernous hemangioma of the pterygopalatine fossa in a 65 years old woman. In this case the tumor caused headache and fullness, but audiometry and nasopharyngoscopy were normal^6^ (Table 1).

**Table 1:** Characteristics of the 2 cases of case of cavernous hemangioma of the pterygopalatine fossa.

Author	Age/Sex	Size of the lesion	Symptoms	Management
Sahin B. et al. 2016	65 Female	3.7 × 3 cm	Headache, fullness on the face	Endoscopic endonasal resection of the lesion
The present study	55 Male	3.4 × 2.8 cm	Hearing loss, fullness on left ear	Endoscopic endonasal resection of the lesion

To our knowledge, our case seems to be the first case of recurrent secretory otitis media caused by an hemangioma of the II trigeminal branch of infratemporal and pterygopalatine fossa.

## Conclusion

At the moment, the diagnostic algorhythm of recurrent secretory otitis media includes, besides audiometry, nasopharyngoscopy as the only instrumental tool. This determines, in a small amount of cases, inability to find a definite cause of the symptomatology, leading the physician to assign this disorder to a more generic tubaric dysfunction. Actually, even if rare, serous otitis media may be caused by the presence of cranial base masses that determine an *ab extrinseco* Eustachian tube obstruction. In our opinion, it is therefore necessary to add an imaging tool to the diagnostic algorithm, preferably a MRI, in all those cases of secretory otitis media lasting more than 3 months that present a negative nasopharyngoscopy evaluation.

## Conflicts of interest

The authors declare no conflicts of interest.
